# Outcomes of pregnancy-related referrals from rural health facilities to two central hospitals in Harare, Zimbabwe: a prospective descriptive study

**DOI:** 10.1186/s12913-021-06289-4

**Published:** 2021-03-25

**Authors:** William Busumani, Paddington T. Mundagowa

**Affiliations:** 1 Faculty of Medicine and Health Sciences, University of Zimbabwe, Harare, Zimbabwe; 2 Ministry of Health and Child Care, Bulawayo, Zimbabwe; 3 Clinical Research Centre, Africa University, 132 H. Chitepo Street, Mutare, Zimbabwe

**Keywords:** Pregnancy, Referral system, Maternal outcomes, Perinatal outcomes

## Abstract

**Background:**

Between the years 2000 and 2017, the global maternal mortality rate dropped by 38% however, 94% of maternal deaths still emanated from low-to middle-income countries. Rural women are at a significantly higher risk of dying from pregnancy when compared to their urban counterparts. Early detection of complications and prompt referral to higher levels of care can reduce the associated maternal and perinatal mortality. This study aimed to determine the maternal and perinatal outcomes of pregnancy-related referrals from rural health facilities to central hospitals in Harare, Zimbabwe.

**Methods:**

A prospective descriptive study was conducted using a sample of 206 patients. All mothers who were referred from rural healthcare facilities were recruited for participation. Data were extracted from patient notes using a structured questionnaire and missing information was obtained from the mother after she had recovered. Bivariate analysis was done using IBM SPSS.

**Results:**

The average age of study participants was 27.4 ± 7.7 years. 87.4% had booked for antenatal care and 81.6% presented to the tertiary facility with their referral notes. The major reasons for referring patients were previous cesarean section (20.4%) and hypertensive disorders in pregnancy (18.4%). There were nine maternal deaths thus a case fatality rate of 4.4% while the perinatal mortality rate was 151/1000 live births. Young mothers were at a higher risk of having adverse perinatal outcomes while primiparous mothers were more likely to have a blood transfusion. Mothers who traveled for > 100 km to the tertiary facility and those who did not attend any antenatal visit were more likely to need blood transfusion. Delivering at the rural health facility was significantly associated with receiving a blood transfusion at the tertiary facility. Mothers who did not attend antenatal visits were more likely to have negative perinatal outcomes.

**Conclusion:**

The proportion of obstetric patients being referred from rural facilities to tertiary institutions for complications reveals how primary and secondary healthcare facilities in Zimbabwe are falling short of offering the services they should be offering. Equipping these facilities with skilled human resources as well as contemporary equipment could help decongest the central hospitals consequently reducing the adverse maternal and perinatal outcomes.

## Background

Despite the fall in the global maternal mortality ratio (MMR) by nearly 44% from an average MMR of 385 maternal deaths per 100,000 live births in 1990 to an estimated average of 216 maternal deaths per 100,000 live births in 2015 [1], the global maternal death rate is still unacceptably high. While the world celebrated a 38% drop in MMR between the years 2000 and 2017, the low and lower-middle-income (LMICs) were still reeling under a disproportionate burden of shouldering 94% of all global maternal deaths [2]. The differences in regional maternal death rates reflect inequalities in access to health services and reveal the underlying gap between the rich and the poor.

Although most of the pregnancy and childbirth complications are unpredictable, they are preventable or treatable. Nearly 75% of all maternal deaths can be attributed to severe bleeding (post-childbirth), infections (post-childbirth), preeclampsia and eclampsia, complication of delivery as well as unsafe abortions [3]. High maternal and perinatal mortality rates in LMICs can be attributed to three delays [4]. The first delay entails the time taken to recognize danger and making the decision to seek care, the second delay includes time taken in reaching the appropriate facility and the third delay is the time taken in receiving quality care once the woman reaches the healthcare facility. A functional referral system is critical in addressing the second and third delays.

The capacity to deal with the unpredictable complications of pregnancy and childbirth at the primary level of care is limited due to the lack of skilled human resources and facilities hence the need to refer patients to higher levels of care. Key requisites for successful maternity referral systems in developing countries include a referral strategy informed by the assessment of population needs and health system capabilities, a well-resourced referral center, good collaboration between referral levels, formalized communication, and transport arrangements between peripheral and referral centers. Furthermore, an effective referral system has agreed upon area-specific protocols for referrer and receiver, supervision and accountability for provider performance, affordable service costs, the capacity and ability to monitor effectiveness, and policy support [5, 6].

Rural-urban health inequities result from weaker health systems and adverse social and environmental determinants experienced by the poor living in rural areas [7]. A study conducted in Zambia reported that women from remote and poorest districts of the country only attended a single antenatal clinic (ANC) visit even when the facilities were closer to their homes because of inadequate staff and low quality of services at these facilities [8]. The same study also cited that women’s livelihoods such as the nomadic lifestyles and household chores influenced maternal decision to go for subsequent ANC attendance after the first visit. Another study noted that punitive measures imposed on women by the health workers and/or incentives provided by the nongovernmental organizations prompted women to attend ANC only once [9].

In 2017, Zimbabwe was ranked among the 15 countries that were considered to be ‘very high alert’ or ‘high alert’ with MMRs ranging from 31 to 1150 on the Fragile States Index [2]. Maternal mortality in Zimbabwe remains pervasive and some women opt for community delivery due to negative labels attached to health facilities [10]. Such labels include exorbitant costs of services, poor attitudes of health providers, extended waiting times, and geographical barriers like long distances between residences and health facility. Rural women are at a significantly higher risk of dying from pregnancy when compared to women living in urban settings [11, 12]. The MMR in Zimbabwe shows an intolerably high and oscillating trend from 694 maternal deaths per 100,000 live births in 1999 [13], 729 in 2009 [14], 614 in 2014 [15] to 650 in 2016 [16]. The fluctuating national trend suggest a deficiency of sustainable solutions to the problem which is most prominent in rural settings.

The rural health center offers the basic emergency obstetric care package and is the first line of healthcare services for the pregnant woman. In Zimbabwe, all eight rural provinces have provincial hospitals which are supported by district and mission hospitals as well as primary care facilities. According to the World Health Organization (WHO) levels of obstetric care, the district, provincial and central hospitals are all level 2 health centers, and these act as referral centers for the primary healthcare facilities. Besides referrals from rural institutions, the central hospitals in Harare also has urban health facilities that refer complicated obstetric cases, but for this study, only referrals from the rural provinces were considered.

The majority (67%) of Zimbabweans live in rural areas [17] where it is difficult to access and afford healthcare services. Approximately 23% live within 5 to 10 km and 17% reside more than 10 km from the nearest health center [14] while some have to walk between 10 and 50 km to access the nearest health facility [18]. The deficiencies of professional skills, medical supplies, and equipment particularly in the rural parts of the country limit the health workers’ options to provide care and treatment [19, 20], thus the need to refer complicated cases to higher levels of care.

Rural healthcare facilities in the Northern Region of the country refer their complicated pregnancy-related cases to two central hospitals namely Harare Central Hospital and Parirentyatwa Group of Hospitals. A preliminary review of maternal mortality registers revealed that during the first quarter of 2017, Parirenyatwa Group of Hospitals recorded a total of 24 maternal deaths and 13 (54%) of these were patients referred from rural health facilities outside the city of Harare. Although there are guidelines to regulate the referral pathway for the potentially life-threatening pregnancy-related complications, this channel was poorly utilized in Zimbabwe with little to no information being given on communicating transfers and giving feedback. A local study on the effectiveness of the referral system for antenatal and intrapartum problems reported that health professionals failed to comply with the referral system recommendations [21]. Thus, there is limited empirical evidence on the outcomes of pregnancy-related referrals from rural health facilities to various tertiary facilities. It was also not clear whether referring obstetric patients from primary health centers, district, and provincial institutions can improve the pregnancy-related outcomes.

The quality of obstetric care provided by facilities can be assessed using either process indicators like the referral system or outcome indicators like the maternal mortality ratio [22]. This study aimed to determine the outcomes of pregnancy-related referrals to Harare Central Hospital and Parirenyatwa Group of Hospitals from rural health facilities outside Harare. Establishing the outcomes of the pregnancy-related referrals would help in understanding the lived experiences of patients on emergency referrals to the two central hospitals targeting improving and sustaining the referral pathway while enhancing pregnancy outcomes.

## Methods

### Research design

This was an open prospective descriptive study that used a quantitative design.

### Study setting

Zimbabwe has 10 administrative provinces (eight rural and two urban). The eight rural provinces have 64 administrative districts and 44 district hospitals. According to the Zimbabwe National Health Strategy (2016–2020), there were a total of 1429 primary health centers in 2015. Referring patients to the central hospitals is based on a zoning system with Harare Central Hospital receiving referrals from Mashonaland East Province while Parirenyatwa Group of Hospitalsreceives referrals from Mashonaland West and Central Provinces. These two tertiary institutions are the busiest public health service centers in Zimbabwe. High-risk pregnancies identified at district and provincial hospitals and cannot be managed at that level are referred to the two tertiary hospitals for further care.

### Study population

The study population was made up of an exhaustive sample of all women who were beyond 20 weeks pregnant and up to 6 weeks post-delivery or miscarriage regardless of the pregnancy outcome. These women were referred to the two central hospitals from a rural health facility during the study period. The enrolled women presented at the two central hospitals for management of complications of pregnancy, labor, delivery, or puerperium. Although the two central hospitals used as study sites in this study also received patients from the public and private health institutions within Harare, study participants were mothers referred to the two central hospitals from the surrounding rural health centers during the period 1 December 2017 to 28 February 2018. Thus, women referred from health facilities within Harare were excluded. Depending on the reason for referral, the mother-baby dyad whose information was used in this study wasstable, seriously or terminally ill, or deceased.

### Sample size and sampling procedure

According to a study on the effectiveness of maternal referral system in a rural setting in Tanzania, 12% of women were referred to a tertiary hospital due to obstetric historical risks [23]. Using Fisher’s formula and assuming that 12% of women were referred due to obstetric historical cesarean section, at a 95% confidence interval and a level of significance of 0.05, the calculated minimum number of participants was 163. The researchers used a census of all the women that presented at the two central hospitals as referrals from rural healthcare centers.

### Data collection and analysis

The study participants were identified by reviewing all women admitted at the obstetrics and gynecological units at the two central hospitals. A structured questionnaire was used to extract information from the patient’s registers as well as case notes while missing details were obtained from the health workers who accompanied or were managing the patient. If some of the information was still missing, it was sought from the survivors after they had been stabilizedand certified to be fit for interviewing by their physician. This was done by the principal investigator and two trained research assistants. Confidentiality was maintained by using patient codes and the database was kept personal password-protected computer only accessible to the principal investigator.

Data were entered and cleaned using Microsoft Excel before it was exported IBM SPSS statistical data editor version 20 for analysis. Frequency and crosstables were produced for the demographic and clinical variables as well as for the underlying causes. The researchers conducted the bivariate analysis at a 95% confidence interval for factors potentially associated with pregnancy-related referrals using need for blood transfusion, need for ICU admission, perinatal outcomes and maternal outcomes as dependent variables.

Written informed consent was sought from all study participants and if the participant deceased on the way to the hospital or died before recovering, consent was obtained from the surviving spouse or the hospital medical superintendent. All obtained information was treated with confidentiality. Permission to carry out the study was sought and obtained from Harare Central Hospital and Parirenyatwa Group of Hospital authorities while ethical clearance was obtained from The Joint Research Ethics Committee and Medical Research Council of Zimbabwe (MRCZ/B/1378).

### Definition of terms

#### Booked pregnancy

Pregnant women registered for follow-up pregnancy-monitoring visits at a particular health facility.

#### Stillbirth

A baby having no signs of life at birth.

#### Early neonatal deaths

Deaths occurring during the first 7 days of life.

#### Maternal death

A death that occurred at any time between admission for labor and delivery and day 42 post-partum.

#### Referral

Transfer of a woman from a rural healthcare facility (primary care center, district hospital, or provincial hospital) to which she was booked or admitted for labor and delivery to a facility providing a higher level of care which was a central hospital.

## Results

### Demographic characteristics

A total of 206 women referred to the two tertiary hospitals in Harare from surrounding rural health facilities with pregnancy-related complications were recruited. The mean age of the women was 27.4 ± 7.7 years. The mean years spent in formal education was 9.2 ± 3.3 years and the median parity was 1. Table 1 shows the demographic profile of the study participants.

**Table 1:** Socio-demographic characteristics of the mothers referred from a rural health care facility to the central hospital

Variable	Demographic characteristics	Frequency, n(%)
Age group (completed years)	< 20	42(20.4)
	20–34	121(58.7)
	> 34	43(20.9)
Marital status	Single	13(6.3)
	Married	189(91.7)
	Widowed	2(1.0)
	Divorced	2(1.0)
Religion	Pentecostal	155(75.2)
	Apostolic	45(21.8)
	Traditional	5(2.4)
	Muslim	1(0.5)
Parity	0	69(33.5)
	≥1	137(66.5)
Formal education (years in school)	0	13(6.3)
	1–7	47(22.8)
	8–11	133(64.6)
	> 11	13(6.3)

### Characteristics of referrals

Approximately 38.3% (*n* = 79) of women were referred during the antenatal period, 41.3% (*n* = 85) during intrapartum period, 18% (*n* = 37) during the post-partum period while 2.4% (*n* = 5) had a miscarriage. Most of the women 87.4% (*n* = 180) had booked their pregnancies and the median ANC visits was 2 (SD = 0.95). 61.7% (*n* = 127) delivered by cesarean section. 15.5% (*n* = 32) required blood transfusion, while 5.3% (*n* = 11) required intensive care unit (ICU) admission. Most women, 78.6% (*n* = 162) delivered at the two central hospitals, while 16.5% (*n* = 34) had delivered either at the district or provincial hospital while 3.9% (*n* = 8) delivered at home.

More than half of the referrals, 55.8% (*n* = 115), were from Mashonaland West Province while 27.2% (*n* = 56) were from Mashonaland East Province, 10.7% (*n* = 22) from Mashonaland Central Province, and 6.3% (*n* = 13) were from other provinces. After stratifying according to referring districts, a total of 27 districts referred the women with suspected pregnancy complications to either Harare Central Hospital or Parirenyatwa Group of Hospitals with the Mashonaland West Province’s Chegutu 19.9% (*n* = 41) and Zvimba 14.6% (*n* = 30) Districts having the majority of referrals. 61.2% (*n* = 126), 30.1% (*n* = 62) and 3.4% (*n* = 7) were referred by a doctor, midwife and clinical officer, respectively. About 3.4% (*n* = 7) were referred by a registered nurse and 1.9% (*n* = 4) were self-referrals. More than four-fifths of the patients (81.6%, *n* = 168) had referral notes while two-thirds (61.7%, *n* = 127) traveled more than 100 km from the referral centers with 24.3% (*n* = 50) traveling for more than 2 h to arrive at the central hospital. Most women, (88.3%, *n* = 188) experienced no delay. The most significant delay was the first delay, (*n* = 10) followed by the third delay, (*n* = 8). The third delay was mostly caused by lack of blood, (*n* = 4) and lack of skills on the part of the health worker (*n* = 4).

### Reasons for referring patients

Table 2 shows the reasons for being referred from the rural facility to the central hospital.

**Table 2:** Top ten reasons for referral

Reason for referral	Frequency, n (%)
Previous cesarean section	42(20.4)
Hypertensive disorders of pregnancy	38(18.4)
Obstructed labor	20(9.7)
Mal-presentation	14(6.8)
Antepartum hemorrhage	12(5.8)
Obstetric sepsis	11(5.3)
Post-partum hemorrhage	8(3.9)
Eclampsia	7(3.4)
Post-dates	6(2.9)
Fetal distress	6(2.9)
Other	42(20.4)

### Maternal and perinatal outcomes

Maternal morbidity was caused by hypertensive disorders (6.8%, *n* = 14), sepsis (5.8%, *n* = 12), anemia (5.3%, *n* = 11) and vesico-vaginal fistula (1%, *n* = 2). There were nine maternal deaths recorded during the study period and the case fatality rate (CFR) was 4.4%. Maternal mortality was caused by post-partum hemorrhage (*n* = 2), eclampsia (*n* = 2), post-abortal sepsis (*n* = 1), ectopic pregnancy (*n* = 1), peri-abortal hemorrhage (*n* = 1), ruptured uterus (*n* = 1) and advanced breast cancer (*n* = 1). Fetal outcomes showed that 82.6% (*n* = 170) were livebirths, 10.7%, (*n* = 22) were stillbirths and 3.4% (*n* = 7) were early neonatal deaths. Thus, the perinatal mortality rate was 151/1000 live births.

### Factors associated with outcomes of pregnancy-related referrals from rural health facilities to two central hospitals in Harare

A chi-square test for association was performed between the outcome variables (need for blood transfusion, ICU bed as well as maternal and perinatal outcomes) and independent variables (socio-demographic information and health system-related factors). Table 3 shows the socio-demographic factors associated with pregnancy-related outcomes while Table 4 shows the health system-related factors associated with pregnancy-related outcomes.

**Table 3:** Sociodemographic factors associated with pregnancy-related outcomes

Variable	Characteristic	Need for blood transfusion		OR (95% CI)	*p*-value
		**Yes (*n*** **= 32)**	**No (*n*** **= 174)**		
Age (completed years)	< 20	10	32	2.07 (0.87–4.67)	0.10
	> 20	22	142		
Marital status	Married	31	158	3.14(0.40–24.55)	0.28
	Unmarried	1	16		
Parity	0	18	51	3.10 (1.43–6.70)	0.004*
	> 1	14	123		
Formal education (years in school)	0–7	4^a^	56	0.30 (0.10–0.90)	0.03
	> 7	28	118		
Religion	Apostolic	8	37	1.23 (0.51–2.97)	0.64
	Other	24	137		
		**Needed Intensive care admission**			
		**Yes (*n*** **= 11)**	**No (*n*** **= 195)**		
Age (completed years)	< 20	2	40	0.86 (0.18–4.14)	0.85
	> 20	9	155		
Marital status	Married	10	179	0.89 (0.11–7.43)	0.92
	Unmarried	1	16		
Parity	0	5	64	1.71 (0.50–5.80)	0.39
	> 1	6	131		
Formal education (years in school)	0–7	8	52	7.33 (1.87–28.70)	0.004
	> 7	3^a^	143		
Religion	Apostolic	1	44	0.34 (0.043–2.76)	0.31
	Other	10	151		
		**Perinatal outcomes**			
		**Dead (*n*** **= 46)**	**Alive (*n*** **= 160)**		
Age (completed years)	< 20	19	23	4.19 (2.01–8.74)	0.0001*
	> 20	27	137		
Marital status	Married	40	149	0.49 (0.17–1.41)	0.19
	Unmarried	6	11		
Parity	0	17	52	1.22 (0.61–2.41)	0.57
	> 1	29	108		
Formal education (years in school)	0–7	10	50	0.61 (0.28–1.33)	0.21
	> 7	36	110		
Religion	Apostolic	12	33	1.36 (0.63–2.91)	0.43
	Other	34	127		
		**Maternal outcomes**			
		**Dead (*n*** **= 9)**	**Alive (*n*** **= 197)**		
Age (completed years)	< 20	2	40	1.12 (0.22–5.61)	0.89
	> 20	7	157		
Marital status	Married	3^a^	14	6.82 (1.54–30.22)	0.01
	Unmarried	6	191		
Parity	0	3	66	0.99 (0.24–4.09)	0.99
	> 1	6	131		
Formal education (years in school)	0–7	5	55	3.23 (0.84–12.46)	0.09
	> 7	4	142		
Religion	Apostolic	1	44	0.43 (0.053–3.57)	0.44
	Other	8	153		

*p-value < 0.05, result is statistically significant; ^a^cell contents less < 5, Chi-square test may not be valid; *OR* Odds Ratio, *CI* Confidence interval

**Table 4:** Health system factors associated with pregnancy-related outcomes

Variable	Characteristic	Need for blood transfusion		OR (95% CI)	*p*-value
		**Yes (*n*** **= 32)**	**No (*n*** **= 174)**		
Reasons for referral	Previous C/S	5	37	0.75 (0.27–2.08)	0.58
	Other	27	137		
Duration of the journey (hours)	> 2	11	39	1.81 (0.81–4.08)	0.15
	< 2	21	135		
Distance (km)	> 100	27	100	4.0 (1.47–10.87)	0.007*
	< 100	5	74		
Stage of referral	Partum (Intra and post)	18	104	0.97 (0.44–2.13)	0.93
	Antenatal	12	67		
Antenatal booking	No	8	18	2.89 (1.13–7.37)	0.03*
	Yes	24	156		
Place of delivery	Other	12	32	2.66 (1.18–6.0)	0.018*
	Central hospital	20	142		
		**Needed Intensive Care admission**			
		**Yes (*n*** **= 11)**	**No (*n*** **= 195)**		
Reasons for referral	Previous C/S	2^a^	40	0.78 (0.16–3.76)	0.76
	Other	9	155		
Duration of the journey (hours)	> 2	4	46	1.85 (0.52–6.61)	0.34
	< 2	7	149		
Distance (km)	> 100	8	119	1.70 (0.44–6.62)	0.44
	< 100	3	76		
Stage of referral	Partum (Intra and post)	9	118	2.94 (0.62–13.96)	0.18
	Antenatal	2	77		
Antenatal booking	No	1	25	0.68 (0.083–5.54)	0.72
	Yes	10	170		
		**Perinatal outcomes**			
		**Dead (*n*** **= 46)**	**Alive (*n*** **= 160)**		
Reasons for referral	Previous C/S	11	31	1.31 (0.60–2.86)	0.50
	Other	35	129		
Duration of the journey (hours)	> 2	13	37	1.31 (0.63–2.74)	0.47
	< 2	33	123		
Distance (km)	> 100	34	93	2.04 (0.98–4.23)	0.055
	< 100	12	67		
Stage of referral	Partum (Intra and post)	24	98	0.89 (0.44–1.80)	0.75
	Antenatal	17	62		
Antenatal booking	No	10	16	2.5 (1.05–5.97)	0.04*
	Yes	36	144		
Place of delivery	Other	17	27	2.89 (1.39–5.98)	0.0043*
	Central hospital	29	133		
		**Maternal outcomes**			
		**Dead (*n*** **= 9)**	**Alive (*n*** **= 197)**		
Reasons for referral	Previous C/S	5	37	5.41 (1.38–21.11)	0.02
	Other	4^a^	160		
Duration of journey (hours)	> 2	2	48	0.89 (0.18–4.41)	0.88
	< 2	7	149		
Distance (km)	> 100	6	121	1.26 (0.31–5.17)	0.75
	< 100	3	76		
Stage of referral	Partum (Intra and post)	2	120	0.25 (0.047–1.30)	0.1
	Antenatal	5	74		
Antenatal booking	No	5	21	10.48 (2.61–42.08)	0.0009
	Yes	4^a^	176		
Place of delivery	Other	4	40	3.14 (0.81–12.23)	0.1
	Central hospital	5	157		

*p-value < 0.05, result is statistically significant; ^a^cell contents less < 5, Chi-square test may not be valid; *OR* Odds Ratio, *CI* Confidence interval, *C/S* Cesarean section

## Discussion

The role of modern maternity care is to ensure safe maternal and fetal outcomes during childbirth. This study sought to determine the outcomes of pregnancy-related referrals to Harare Central Hospital and Parirenyatwa Group of Hospitals from rural health facilities outside the city of Harare. Understanding the characteristics of referrals, the current operation of the referral pathway could be used to craft interventions towards the reduction of maternal and perinatal mortality in the area under study.

A maternal age of less than 20 years was associated with adverse perinatal outcomes (mortality), a finding that was similar to other studies [24, 25]. This could be due to failure to achieve the minimum number ANC visits (four) as a result of inadequate knowledge on the essential aspect of these visits by the adolescent mothers [26]. The average number of ANC visits in this study was two. Teenage pregnancies are usually unplanned and unwanted [27] hence the young mother may have increased psychosocial stress to focus on pregnancy monitoring and the possible outcomes of the pregnancy.

This study revealed that low parity was associated with the need for obstetric blood transfusion. This result was contrary to findings by Eyelade and colleagues who reported that the need for blood transfusion rates increased with increasing parity [28]. The difference could be due to the latter study using a larger sample size when compared to the one used in this study. However, a study on risk factors for blood transfusion in Finland noted that primiparity predisposed women to blood transfusion [29]. Low parity which is usually associated with younger age, can result in increased blood loss due to tearing of the premature tissues of the birth canal [30].

The literature review showed varying rates and reasons for referral of obstetrics patients from peripheral to tertiary health facilities [31, 32]. This is mainly due to the different clinical criteria used for transferring and admitting patients, as well as distance traveled [33]. It was noted that the majority of participants in this study delivered by cesarean section at the two central hospitals despite having provincial and district hospitals which are supposed to be equipped to carry out the procedure. Ghardallou and colleagues also observed that lower level health facilities in Tunisia still referred patients with obstetric complications in spite of the improvement of peripheral facilities. This could be attributed to poor technical ability [5], lack of qualified staff resulting in the poor quality of care at the peripheral health care center, and poor quality of antenatal follow-up [34]. About 81.6% of the referred patients had referral notes, a percentage higher than findings by Moakoh-Coleman et al. (2019) in the Greater Accra Region in Ghana where they cited that 37.8% of the referred patients had referral notes [35]. The high proportion of the availability of referral notes in this study is commendable although it can still be improved by giving feedback to lower levels of care. It is worth noting that this study did not investigate the completeness of the referral notes which is a key component towards the provision of comprehensive care. Future scientific inquiries into the completeness of referral notes can aid in determining whether the referring health workers understand the operations of the referral system.

The proportion of stillbirths in this study was high (10.7%) when compared to findings from a Tunisian study (1.8%) [33] but lower when compared to a similar study in Nigeria (16.4%) [36]. This is a reflection of the deteriorated healthcare services in Zimbabwe and other sub-Saharan countries which persistently report low or stagnant coverages of effective interventions recommended for maternal, neonatal, and child health [37]. Contrary to Patel et al.’s reported findings in which most of the pregnancy–related referrals were due to obstructed or prolonged labor [38], this study noted that the major reasons for transferring patients were previous cesarean section and hypertensive disorders of pregnancy. Referring patients with previous cesarean section was mainly due to an understanding that a third to half of cesarean surgeries are performed due to a history of cesarean delivery [39, 40]. This type of surgery cannot be performed at the primary and most secondary healthcare facilities in Zimbabwe due to the lack of skilled staff and equipment. Hypertensive disorders were also found to be the major causes of referral in other studies [41–44]. The researchers of the present study noted that 38% of the women were referred during antenatal, 41.3% during the intrapartum, and 18% during the post-partum periods. An intrapartum transfer of 41.3% was high when compared to other studies in better developed countries like New Zealand (12.6%) [32], Australia (13.2%) [45], Denmark (11.6%) [46] and England (between 16.7 and 20%) [47]. Developing countries often experience a dearth of fundamental skills and equipment needed for early diagnosis of pregnancy complications and in some cases, these services could be out of reach particularly for the economically disadvantaged living in the rural areas. However, the proportion of women who were referred during the post-partum phase (18%) compared most closely with other studies which reported (16.4%) [46], (16.8%) [45] and (19.6%) [48].

Timely antenatal booking can ensure early identification and appropriate management of mothers at risk or with complicated pregnancies. The present study showed that 87% of the referred patients had been registered for pregnancy monitoring with a health facility, a proportion higher than findings of a similar study in India where they noted that 72% of the referred pregnant women were booked [38]. The WHO recommends that all pregnant women should be booked. Our ANC booking findings compared well with another local study which reported a high ANC attendance rate of 94% by women in rural Gutu District, Zimbabwe [49]. The high number of pregnancy booking could be mainly due to the versatile programs by the Ministry of Health and Child Care and non-governmental organization partners to promote free ANC booking and institutional deliveries performed by a skilled birth attendant in Government rural clinics. The emphasis on the importance of booking and attending ANC visits was highlighted in this study by having mothers who did not attend ANC being 2.5 times more likely to have negative perinatal outcomes. The messages targeting safe motherhood should therefore be sustained if the maternal and perinatal mortality rates are to be reduced in Zimbabwe.

In Zimbabwe, the rural districts have a cesarean section rate of 2.2% [50] which is way below the 5–15% recommended by the WHO [51]. Contrary to the low cesarean section rates in rural Zimbabwe, urban districts met the WHO minimum cesarean section targets of 5% . Approximately 61.7% delivered by a cesarean section which is higher than 32.7% reported in a similar study [52]. It is important to note that most cases traveled long distances to access obstetric care at the two central hospitals thus, their condition may have deteriorated along the way prompting the need for urgent obstetric intervention. Low cesarean section and operative delivery rates in rural areas could also be attributed to lack of trained personnel and possible reluctance to perform vacuum deliveries thus leading to high perinatal mortality rates [52]. Obstetric hemorrhage is a life-threatening condition with post-partum hemorrhage being the most common cause of maternal morbidity and mortality [53]. Our study findings showed that 15.5% of the mothers who were transferred to Harare Central Hospital and Parirenyatwa Group of Hospitals required blood transfusion. This was slightly elevated when compared with a Nigerian study which reported an overall obstetric blood transfusion rate of 12.1% [54]. The difference in blood transfusion rates could be explained by having the present study focusing on urgent complicated obstetric cases observed over a shorter period of 3 months while Anorlu and others conducted their study over a three-year period. Mothers who traveled more than 100 km were 4 times more likely to be transfused than those who traveled fewer kilometers and mothers who delivered at the rural facility or on transit were 2.66 times more likely to be transfused. Nearly two-thirds of the referred patients had a cesarean section which is a procedure that carries a risk of major intra-operative blood loss because the majority of maternal deaths occur as a consequence of post-partum hemorrhage associated with cesarean section [55].

The present study revealed that 5.3% required ICU admission for hemorrhage or hypertensive related complications. This proportion was higher than the one documented in a study by Jamal et al., 2018 (1.3%) [56] and the higher rates observed in this study may be due to the persistent limited supply of blood for transfusion at primary and secondary health care centers in Zimbabwe hence they transfer critically ill patients to tertiary care facilities.

There were nine maternal deaths (CFR = 4.4%) during the 3 months of the study period and this frequency was very high when compared to other studies which reported no deaths over 3 months [33] and 13 deaths over 4 years [38]. UNICEF/WHO/UNFPA recommend a maximum acceptable CFR of less than 1% [57]. The CFR in this study was higher than the recommended level because some patients traveled for long distances to reach the central hospitals and this may also be due to the three delays. The perinatal mortality rate was 151/ 1000 live births which was high when compared to findings from a similar study in India (95.23/1000 live births) [44]. Traveling long distances can increase the chances of fetal distress thereby increasing the possibility of intrauterine death. However, this study did not ascertain the actual obstetric events leading to maternal and perinatal deaths and investigators recommend future inquiry into these two public health challenges.

When compared with urban pregnant women, mothers who dwell in rural regions often face considerable limitations when it comes to utilization of obstetric care services. Many developing countries do not have resources to fully equip rural facilities at the same extend as the urban facilities [58]. Furthermore, there is little demand for equal access for emergency obstetric care by the rural women due to limited education when compared to urban women. Geographical accessibility and transport availability are often poor in rural areas when compared to urban setting where private and public transport is available and sufficiently affordable to mothers seeking health care. High population densities in urban areas also enhance geographical proximity of healthy facilities when compared to the sparsely populated rural settings [58].

### Limitations

The present study only included those women who were referred from rural facilities and managed to arrive at the central hospitals. Women who were referred but failed to arrive at the central hospitals because of failure to secure transport or dying in transit were not included thus, the number of such patients is not known. Interviewing the health workers at primary and secondary facilities could have exposed the actual reasons for referring patients and assisted in relating these to the maternal outcomes but this was not done due to financial constraints on the part of researchers. The authors recommend a follow-up qualitative study to understand the health workers’ perspective on the operations of the patient referral system.

## Conclusion

Obstetric complications of maternal referrals from rural facilities to the central hospitals were associated with adverse maternal and perinatal outcomes. The proportion of obstetric patients being referred to tertiary institutions reveals how primary and secondary healthcare facilities in Zimbabwe are falling short of offering the services they should be offering. Equipping these facilities with skilled human resources as well as contemporary equipment could help decongest the central hospitals consequently reducing the maternal and perinatal mortality rates caused by delays that occur when the patients are transferred to higher levels of care.

## Data Availability

The datasets used and/or analyzed during the current study are available from the corresponding author on reasonable request.

## Abbreviations

ANC: Antenatal Clinic
CFR: Case fatality rate
ICU: Intensive care unit
LMICs: Low and middle-income countries
MMR: Maternal mortality rate
WHO: World Health Organization
